# Efficient separation of butane isomers via ZIF-8 slurry on laboratory- and pilot-scale

**DOI:** 10.1038/s41467-022-32418-6

**Published:** 2022-08-15

**Authors:** Mingke Yang, Huishan Wang, Julian Y. Zuo, Chun Deng, Bei Liu, Liya Chai, Kun Li, Han Xiao, Peng Xiao, Xiaohui Wang, Wan Chen, Xiaowan Peng, Yu Han, Zixuan Huang, Baocan Dong, Changyu Sun, Guangjin Chen

**Affiliations:** 1 State Key Laboratory of Heavy Oil Processing, China University of Petroleum, Beijing, 102249 China; 2 FMG Inc., Edmonton, AB T6N 1M9 Canada; 3 CenerTech Tianjin Chemical Research and Design Institue Co., Ltd., Tianjin, 300131 China

**Keywords:** Chemical engineering, Natural gas, Chemical engineering, Metal-organic frameworks

## Abstract

n-butane and isobutane are important petrochemical raw materials. Their separation is challenging because of their similar properties, including boiling point. Here, we report a zeolitic imidazolate framework-8 (ZIF-8)/N,N-Dimethylpropyleneurea (DMPU)-water slurry as sorption material to separate butane mixtures. The isobutane/n-butane selectivity of ZIF-8/DMPU-water slurries is as high as 890 with high kinetic performance, which transcends the upper limit of various separation materials or membranes reported in the literature. More encouragingly, a continuous pilot separation device was established, and the test results show that the purity and recovery ratio of isobutane product are 99.46 mol% and 87%, respectively, which are superior to the corresponding performance (98.56 mol% and 54%) of the industrial distillation tower. To the best of our knowledge, the use of metal-organic frameworks (MOFs) for gas separation in pilot scale remains underexplored, and thus this work provides a step forward to the commercial application of MOFs in gas separation.

## Abstract (web summary)

The separation of butane isomers, raw materials in petrochemical industry, is challenging. Here the authors report the separation of n-butane and isobutane using a metal-organic framework slurry; the separation can be performed at large scale in a pilot-scale separation tower.

## Introduction

Both n-butane and isobutane are important petrochemical raw materials used in direct applications or for synthesizing other petrochemicals. The isomers usually coexist as liquefied petroleum gases produced by condensation of natural gas or in oil processing, such as with fluid catalytic cracking (FCC). Hence, it becomes critical to separate butane isomers efficiently and economically. For example, high-purity isobutane can be used for^1^ (1) reactions with olefins to produce alkylated high-octane gasoline, (2) co-oxidation with propylene to coproduce propylene oxide and tert-butanol, and (3) dehydrogenation to produce isobutene. On the other hand, n-butane can be used as a refrigerant or for (1) oxidation to produce maleic anhydride^2^ and (2) dehydrogenation to produce butadiene^3^. However, the separation of butane isomers is considered to be a challenging processes^4,5^ since their physical and chemical properties, such as boiling points, vapour pressures, and polarizabilities, are similar^6^. To date, energy- and cost-intensive distillation techniques are still widely applied for butane isomer separation in the industry owing to a lack of more efficient alternatives.

Adsorptive separation with selective size/shape exclusion provided by inorganic porous materials, such as zeolites and activated carbon, is considered to be an attractive alternative to current energy- and cost-intensive distillation-based separations^7,8^. For example, as the most extensively used agents for butane isomer separation, the MFI-type zeolites (silicalite-1 and ZSM-5) with 5.5 Å multidimensional elliptical pores^9^ can distinguish n-butane from isobutane. Application of MFI zeolites to membrane-based separations has been studied sufficiently^10–13^. The n-butane/isobutane selectivities of MFI membranes tested at 20–100 °C ranged from 4 to 70^5,12^. In addition, Woo et al.^14^ prepared a MFI-based mixed matrix membrane to improve n-butane permeability, but the highest n-butane/isobutane separation factor was only 6.64. In addition to MFI-based membranes, Liu and coworkers^15^ synthesized face-centred cubic (fcu)-type metal-organic framework (MOF) membranes on glassy polymer (6FDA-DAM) supports, which exhibited an n-butane/isobutane separation factor of ~30 at 75 °C. Zhou et al.^5^ prepared high-quality carbon molecular sieving membranes on γ-alumina substrates, and the n-butane/isobutane separation factor reached 74. Nevertheless, even though many studies have claimed effective use of membrane-based separation technologies in butane isomer separations, commercial application has not yet been seen in this field. The reasons are as follows: (1) membrane production is cumbersome and expensive^15^, and membrane structures are prone to cracking^10^. (2) The separation selectivities for linear/branched isomers are low, and the uptake capacities of adsorbents are low^16^. (3) Continuous multistage separation is difficult to realize with membrane-based technology.

MOFs consisting of organic linkers coordinated to metal ions/clusters represent another important category of porous materials, which are very promising candidates exhibiting molecular sieve properties for gas separation^17–20^ due to the relatively easy and flexible tunability of their pore-aperture sizes and structures. Zeolitic imidazolate frameworks (ZIFs) are a subclass of MOFs, and they have attracted extensive attention in the field of gas separation in recent decades^21,22^ due to their excellent thermal and chemical stabilities and large surface areas^23^. For example, the ZIF-8 framework (Zn(mIm)_2_, mIm = 2-methylimidazole), one of the most representative ZIFs, shows excellent performance in CO_2_ capture and light hydrocarbon (including n/iso-paraffins) separation^24–30^. It is worth mentioning that adsorption separation includes two modes^31^: (1) equilibrium separation, in which one component has a higher equilibrium adsorption capacity than the other, and (2) kinetic separation, where one component has a higher uptake rate than the other and selectivity is affected by the separation time. Zhang et al.^32^ reported that ZIF-8 exhibited great kinetic selectivity (2.5 × 10^6^) for n-butane over isobutane by estimating the thermodynamically corrected diffusivities. This value was much higher than the separation factors reported for zeolite-based membranes and MOF-based membranes, which opens an opportunity for using ZIF-8 to separate butane isomers. Then, Zhang and coworkers^33^ successfully increased the n-butane diffusivity of ZIF-8 with post-synthetic thermal modifications. However, to the best of our knowledge, there has been no experimental work on direct separation of butane isomer mixtures by ZIF-8 reported in the literature. Therefore, the actual performance of ZIF-8 in separating n-butane/isobutane mixtures needs further investigation.

In addition, ZIFs, like other powdered porous materials, cannot be used directly for adsorptive separation. One of two emerging technologies is normally used. One option is to mix ZIFs with other materials, such as polymers, to form a mixed matrix membrane for gas separation^34^. Another method is to apply ZIFs in a fixed-bed pressure swing adsorption (PSA) column after shaping-up, but this may significantly reduce the adsorption capacity and speed of the adsorbent^35^. The traditional PSA or TSA (temperature swing adsorption) approaches for the separation of butane isomers may face other challenges. Because butane is relatively heavy, heating is required to achieve complete desorption. However, the thermal conductivity of porous ZIF-8 is low; it is difficult to perform a heating or cooling process in a large-diameter industrial-scale fixed bed column, let alone integrate heating with energy conservation. Hence, ZIFs have not been widely used for industrial gas separations until now. In the past few years, gas-phase simulated moving beds (gas-SMBs) and slurry approaches have emerged as potential alternatives to PSA. Martins et al.^36–38^ explored gas-SMB technology for olefin/paraffin separations and obtained high-purity products and high recovery with continuous countercurrent contact of gas–solid phases. In the slurry approach method, ZIF powder is directly mixed with a suitable solvent to form a flowable fine slurry^39–42^. Compared to the conventional solid adsorption techniques (e.g., fixed-bed PSA or TSA) that suffer from difficult heating, cooling, and heat integration in batch operations, the ZIF slurry flows like a liquid absorbent and can be used in traditional absorption/desorption columns with continuous multistage gas-slurry contact and achieve highly efficient separation, effective heat exchange, and thermal integration. Li et al.^43^ successfully used a ZIF-8 slurry in a pilot-scale packed tower for CO_2_ capture and achieved good separation efficiency, energy consumption, and stability during slurry operation. Pan et al.^44^, Liu et al.^42^, Chen et al.^45^, and Yang et al.^46^ applied this method to separate a series of low boiling gas mixtures, such as natural gases, FCC dry gases, coalbed gases, IGCC syngas, etc. They all achieved promising results.

In this work, we apply the ZIF-8 slurry approach to the more challenging separation of butane isomers. First, we report that an ideal solvent, N,N-dimethylpropyleneurea (DMPU), with low toxicity, low volatility, low viscosity, and high chemical stability, is suitable for preparing ZIF-8 slurries for highly efficient separations of butane isomers. Both single component sorption tests and mixed isomer separation experiments demonstrate that the solvent DMPU significantly increases the sorption speed of n-butane, while water drastically decreases the sorption of isobutane in the ZIF-8/DMPU-water slurry. By optimizing the mass ratio of DMPU to water, high sorption speeds for n-butane and selectivity (>890) for n-butane over isobutane are achieved. Subsequently, column breakthrough experiments are performed to show high kinetic separation performance of the slurry. More encouragingly, an industrial pilot apparatus is established to carry out continuous pilot separation tests, in which the feed gas is a multicomponent butane mixture from a Chinese refinery. To the best of our knowledge, the use of metal-organic frameworks for gas separation on a pilot scale remains underexplored, and thus this work provides a step forward in commercial application of MOFs in gas separation.

## Results

**Fig. 1:**
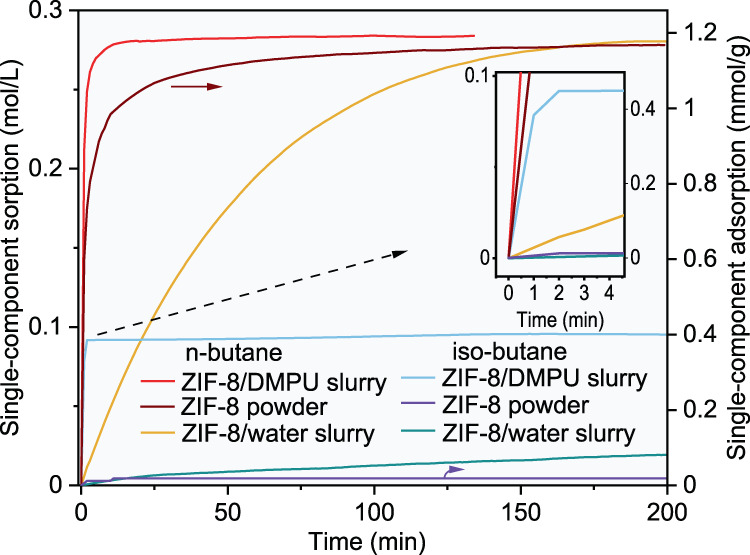
Comparison of the sorption kinetics of single-component in different media. Kinetic profiles of n-butane and isobutane on ZIF-8 powder (right axis), ZIF-8(35 wt%)/DMPU slurry, and ZIF-8(25 wt%)/water slurry (left axis) at 293.15 K and an initial gas-slurry volume ratio of about 6.8 (initial gas–solid volume ratio of about 26.7 for ZIF-8 powder). The profiles were obtained via P-time curves shown in Supplementary Fig. 1.

**Fig. 2:**
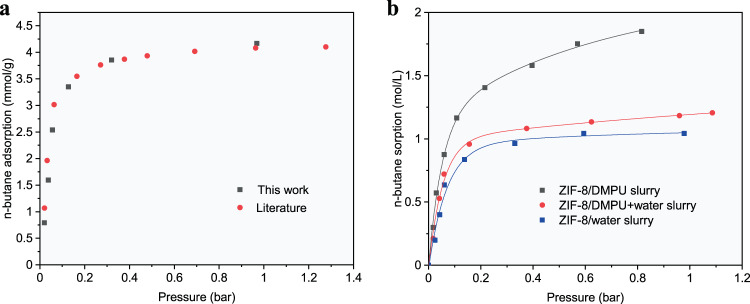
n-butane sorption capacity. Sorption isotherms of n-butane on **a** ZIF-8 powder at 293.15 K measured in this work and the literature^31^, **b** ZIF-8 (35 wt%)/DMPU slurry, ZIF-8 (25 wt%)/water slurry and ZIF-8 (30 wt%) slurry with solvent composition of 80 wt% DMPU + 20 wt% water at 293.15 K. The lines are guides for eyes.

We first tested the behaviours of the ZIF-8 powder and the ZIF-8/water slurry in adsorption of n-butane and isobutane. As predicted by Zhang et al.^32^, our experimental results illustrated in Fig. 1 indicate that very high kinetic selectivities for n-butane over isobutane were achieved with the ZIF-8 powder or the ZIF-8/water slurry. The uptake of isobutane remained very low for a long time, while that of n-butane increased continuously with elapsed time until equilibrium was reached. This is because the –CH_3_ branch in an isobutane molecule hinders entry into the cages of ZIF-8. However, the rate for adsorption of n-butane by the ZIF-8 powder was not high enough for a practical adsorption separation process, although its maximum equilibrium uptake (approximately 4.0 mmol/g as shown in Figure 2a) showed that the n-butane sorption capacity was significantly higher than those of most other adsorption materials reported in the literature, e.g., TIFSIX-3Ni (1.13 mmol/g at 298 K) and ZU-36-CO (2.2 mmol/g at 298 K)^16^, Y-fum-fcu-MOFs (2.0 mmol/g at 293 K)^47^, CMS-PMOF-1 (1.9 mmol/g at 293 K)^48^, ana-ZMOF (0.9 mmol/g at 293 K)^6^, and commercial shaped MFI zeolites, including ZSM-5 (1.1 mmol/g at 300 K)^49^ and silicalite-1 (1.7 mmol/g at 298 K)^12^. The n-butane sorption capacity further decreased when the ZIF-8 powder was shaped for practical PSA or TSA operation (Supplementary Fig. 2). The sorption rate of n-butane in the ZIF-8/water slurry was even lower than that in the solid ZIF-8. Hence, more suitable solvents are needed for preparing ZIF-8 slurries.

**Fig. 3:**
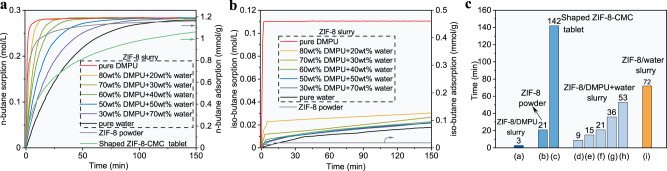
Comparison of the single-component sorption speed of solid ZIF-8 and ZIF-8 slurries. Kinetic profiles of n-butane (**a**) and isobutane (**b**) in ZIF-8 slurries (left axis) and ZIF-8 powder, shaped ZIF-8 (right axis), where temperature, initial gas-slurry volume ratio, initial gas–solid volume ratio and solid ZIF-8 content in the slurries were set to 293.15 K, ~6.8, ~26.7 and 30 wt% respectively. CMC: Carboxymethyl Cellulose Sodium; **c** comparison of time for different sorption media to reach 90% of the equilibrium sorption capacity of n-butane, where “d” to “h” on the abscissa corresponds to 20 to 70 wt% of the water content in the solvent.

**Fig. 4:**
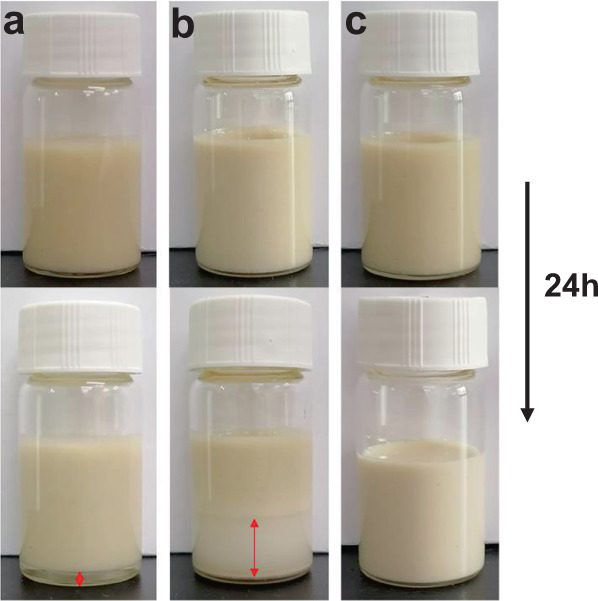
The stability of ZIF-8 slurries. State changes of ZIF-8 (30 wt%) slurry with different liquid media over 24 h: **a** water, **b** solvent DMPU, **c** solvent DMPU (80 wt%) + water (20 wt%). Top: freshly prepared slurries; bottom: slurries after standing for 24 h.

The sorption behaviour of a ZIF-8 slurry strongly depends on the solvent chosen for preparation. The criteria for choosing a suitable solvent are as follows: (1) it cannot enter the pores of ZIF-8; otherwise, the adsorption capacity of ZIF-8 will be lost; (2) the solubility of isobutane in it should be as low as possible; otherwise, the apparent selectivity of the ZIF-8 slurry will be lowered significantly compared with that of solid ZIF-8; hence, it should be hydrophilic; (3) it should be very chemically stable when mixed with ZIF-8 and exhibit low viscosity and volatility; (4) it should accelerate adsorption of n-butane in suspended ZIF-8; and (5) serious foaming should not occur during the desorption process. After a great amount of sieving and testing based on the above criteria, it was found that the DMPU solvent met almost all of the aforementioned requirements, i.e., a much higher sorption speed for the ZIF-8/DMPU slurry than for the solid ZIF-8, as shown in Fig. 1, a high normal boiling temperature, high chemical stability, low viscosity, and minimal foaming during the desorption process, even though there was a certain solubility of isobutane in the slurry. Fortunately, upon adding an appropriate amount of water to the DMPU solvent, the solubility of isobutane decreased drastically; the higher the water concentration was, the lower the solubility of isobutane, as shown in Supplementary Fig. 4. Correspondingly, the sorption capacity of the ZIF-8/DMPU slurry for isobutane was drastically decreased by adding more than 20 wt% water into the solution, as shown in Fig. 3b, which resulted in high selectivity for n-butane over isobutane; this arose because the sorption capacity for n-butane did not decrease significantly upon addition of water, as seen from Fig. 3a, in which the profiles were obtained from the measured P-time curves shown in Supplementary Fig. 3. More importantly, Fig. 3c shows that when the water content in the solution was less than 40 wt%, the overall speed for sorption of n-butane in the slurry remained slightly higher than that seen for the ZIF-8 powder but significantly higher than that for shaped ZIF-8. It should be noted that the sorption speed in a flowable slurry could be further increased by applying mechanical enhancing measures such as stronger stirring, a finer distribution of gas in the slurry or vice versa. At the same time, the n-butane sorption capacity in the ZIF-8 (30 wt%) slurry with a solvent composition of 80 wt% solvent DMPU + 20 wt% water reached 1.2 mol/(L·bar) (Fig. 2b). Another advantage of the ZIF-8/DMPU-water slurry over the ZIF-8/water slurry or the ZIF-8/DMPU slurry was that the ZIF-8/DMPU-water slurry was more stable, while the others separated when left unstirred for certain time periods, as shown in Fig. 4. It is important to keep the slurry uniform in a continuous separation process; otherwise, plugging may occur.

The sorption behaviour of the ZIF-8 slurry also depended strongly on the solid ZIF-8 fraction. Supplementary Fig. 5 shows that both the sorption speed and the equilibrium quantity of n-butane sorbed increased with increasing ZIF-8 mass fraction. However, these increases declined dramatically when the ZIF-8 fraction was higher than 20 wt%. Since a high solid fraction gives rise to high viscosity^46^, a suitable ZIF-8 content is 20–30 wt%.

After determining the suitable solvent for preparing ZIF-8 slurry, a series of separation experiments were performed with gaseous isobutane/n-butane mixtures. The experimental results are tabulated in Supplementary Tables 1–3, in which the pressures *P*_e_, the compositions of the gas phase and slurry (expressed with the dry-basis mole fractions of n-butane, *y*_1_, *x*_1_, respectively), and the selectivities for n-butane over isobutane were determined when the gas-slurry contacting systems became stable, which was marked by a system pressure decay of no more than 5 mbar within 30 min.

**Fig. 5:**
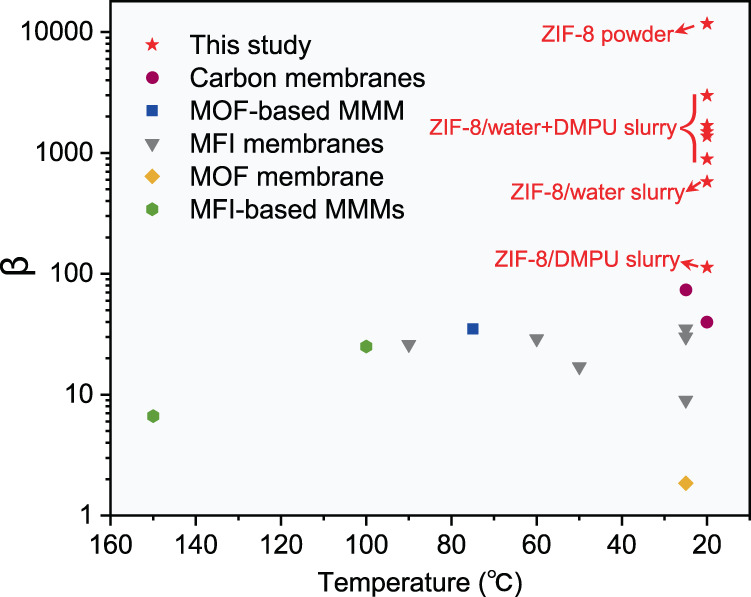
Separation performance of ZIF-8 powder and ZIF-8 slurries compared to top-performing materials. Comparison of the n-butane/isobutane separation factor (*β*) of ZIF-8 powder and ZIF-8 slurries with various membranes reported in literatures. (Carbon memabranes^5,53^; MOF-based mixed matrix membrane^15^; MFI membranes^12,54–58^, MOF membrane^59^ and MFI-based mixed matrix membrane^14,60^).

Supplementary Table 1 shows a comparison of the separation abilities of solid ZIF-8 powder and ZIF-8 slurries with different solvent compositions. As expected, the ZIF-8 powder exhibited the highest selectivity, 11,757, for n-butane over isobutane. Although the selectivity of ZIF-8/water, 577, was high enough, it was much lower than that of the solid ZIF-8 powder because it seemed that isobutane more easily entered the pores of ZIF-8 in the slurry, as indicated by Fig. 1. Compared with the ZIF-8/water slurry, the ZIF-8/DMPU slurry showed larger decreases in separation selectivity; it was only 113 because the solubility of isobutane in the solvent DMPU is much higher than that in water. However, 113 is still higher than all values reported for other separation media, as shown in Fig. 5. Considering the high sorption speed of n-butane in the ZIF-8/DMPU slurry and the fact that multistage separation in an absorption column is easy to realize, a factor of 113 is already high enough for actual application.

Interestingly, the separation selectivity of the ZIF-8/DMPU-water slurry was much higher than those of both the ZIF-8/water slurry and the ZIF-8/DMPU slurry; it ranged from 890 to 2985 when the water content in the mixed solvent was changed from approximately 20 to 70 wt%. More than ~98% of the n-butane was removed from the gas phase after single separation stages in all five experimental runs, and the mole fractions in the gas phase decreased from 38.2 mol% to only approximately 1.35 mol%. These results are all encouraging. As shown in Supplementary Table 1, the separation selectivity increased with increasing water content in the mixed solvent, while the sorption speed decreased with increasing water content, as shown in Fig. 3. Hence, a balance between separation selectivity and sorption speed should be established to achieve more efficient separation of butane isomers. Taking both sorption speed and separation factor into account, we recommend that the most suitable water content in the mixed DMPU-water solvent is 20–30 wt%, and it was set to 20 wt% in subsequent experiments unless otherwise specified. We also tried other slurries prepared with different solvents, such as N,N-dimethylformamide (DMF), 2-methyl-2,4-pentanediol (MPD) and isohexadecane; the relevant experimental results are displayed in Supplementary Fig. 6 and Supplementary Table 4. The separation selectivities for butane isomers in slurries prepared with MPD and isohexadecane were much lower than that for DMPU slurry, although the sorption speeds were higher. In particular, the ZIF-8/isohexadecane slurry showed the highest sorption speed but the lowest separation selectivity, 15, which was much lower than that of the ZIF-8/DMPU slurry, 114. The higher sorption speed mainly resulted from the higher solubilities of butane isomers in solvent. However, the higher solubilities of the butane isomers in the solvent led to a higher total capacity for sorption of isobutane in the slurry, resulting in lower separation selectivity. Additionally, it was found that the chemical stabilities of the slurries prepared with DMF and MPD were low, especially in the presence of water.

Perfect regeneration performance is critical for practical application of a sorbent. Hence, the regeneration performance of one ZIF-8/DMPU-water slurry with a solid content of 30 wt% and solvent composition of 70 wt% solvent DMPU + 30 wt% water was tested. In all experimental runs, the n-butane rich slurry was regenerated by evacuation at 323.15 K for 20 min. These regeneration conditions should be very mild. The experimental results are listed in Supplementary Table 2. During 22 cycles over 14 days, the separation performance of the slurry remained approximately stable, although there were small fluctuations because the feed gas compositions were not fixed perfectly; no tendency for declination was observed. In addition, the XRD diffraction pattern for the ZIF-8 powder recovered after this sorption-desorption cycling experiment was completely consistent with that of fresh ZIF-8 (Supplementary Fig. 7), suggesting the structural integrity of the recovered ZIF-8. The above results indicated that the slurry was perfectly regenerated. In these experiments, the concentration of isobutane in the feed gas was controlled to approximately 90 mol%. One can see that high isobutane purity (>99.6 mol%) was seen in the gas phase after one stage of separation. The separation results obtained for the slurry under different desorption conditions are listed in Supplementary Table 3.

**Fig. 6:**
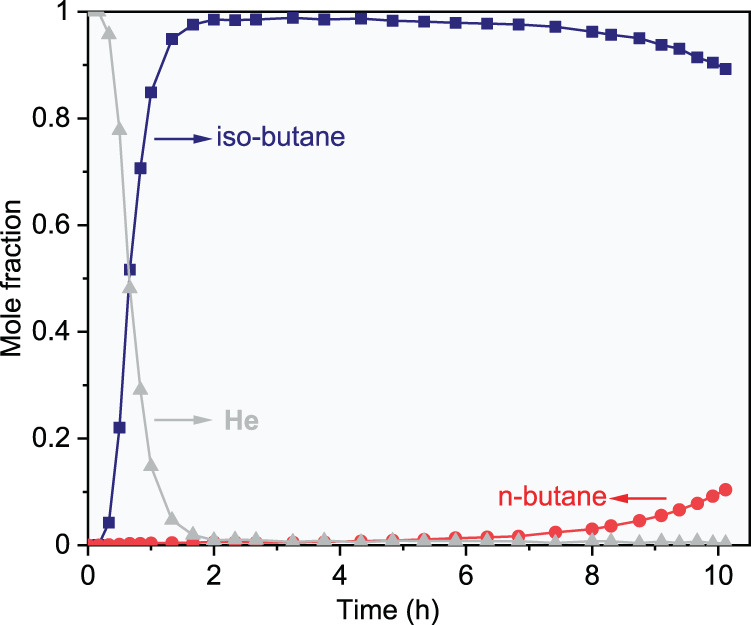
Gas mixture column breakthrough results. Column breakthrough curve for a n-butane(1)/isobutane(2) gas mixture (*z*_2_ = 59 mol%) using ZIF-8(30wt%)/DMPU-water slurry at 303.15 K and 2 bar. (Gas flow rate: 60 mL/min).

The column breakthrough test is an effective tool for evaluating the kinetic separation performance of a sorbent, and it describes the sorption separation process more accurately and evaluates the possibility of using the sorbent in actual industrial processes. Herein, a column (which was filled with 1800 g of ZIF-8 (30 wt%)/DMPU-water slurry) breakthrough test was carried out with an n-butane/isobutane gas mixture (41 mol%/59 mol%) at 303.15 K and 2 bar. As anticipated, excellent separation performance was achieved with the slurry column. As shown in Fig. 6 and Supplementary Table 5, isobutane broke through the column within 10 min and quickly reached 90 mol% within 1.33 h, confirming that the ZIF-8/DMPU-water slurry efficiently excluded isobutane. In contrast, the n-butane concentration remained low for a long time. If He content is not counted, the resulting purity of the isobutane in the outlet gas was maintained at 99+ mol% for 4.83 h; even after 9.92 h, the isobutane concentration in the outlet gas was still higher than 90 mol%. It is worth indicating that the slurries tested in these experiments were used 7 times in 30 days and regenerated with an atmospheric pressure He purge at 353.15 K. The breakthrough test verified the excellent kinetic performance and molecular exclusion effect of the ZIF-8/DMPU-water slurry.

**Fig. 7:**
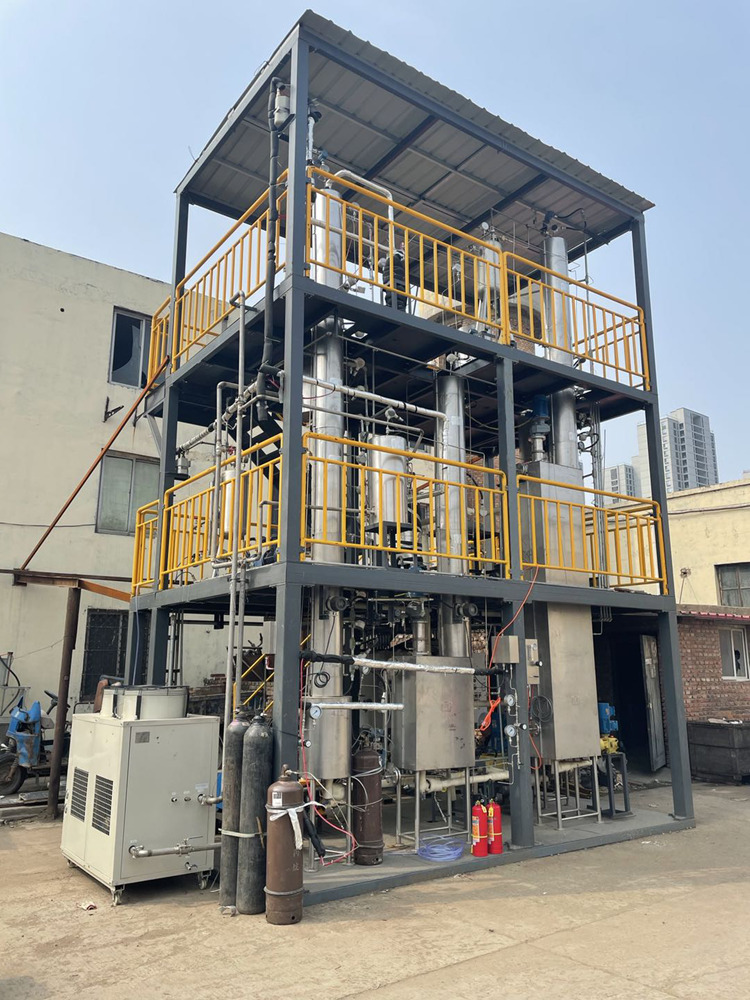
The physical picture of the pilot plant for continuous C4 gas mixture sorption–desorption separation experiment. The main body of the equipment is two packed columns, which are used as absorption column and desorption column respectively, with a height of about 5.5 m.

Furthermore, a C4 continuous separation pilot plant (Fig. 7) was set up to verify the feasibility of the scaled-up slurry separation process. The functional component of the pilot plant was a sorption column (5.17 m) and a desorption column (5.48 m), both of which were placed in CY700 structured packing. We chose a typical multicomponent C4 gas mixture taken from a Chinese refinery as the feed gas. To ensure smooth flow of the slurry, we conservatively set the ZIF-8 content in the slurry at 20 wt% because the lower the ZIF-8 content is, the lower the slurry viscosity. The entire system was charged with 25 kg of ZIF-8 (20 wt%)/DMPU-water slurry. Notably, the ZIF-8 material used in the pilot plant was prepared in large quantity by using a green, low-cost, rapid, and high-yield method on a pilot scale^50^. The test results were very encouraging; representative results and the corresponding operation conditions are shown in Table 1. As seen, the isobutane concentration was effectively enriched from 51.19 mol% in the feed gas to 99.46 mol% in the product gas, while it was only 12.17 mol% in the desorbed gas. After calculation, the recovery ratio for isobutane (*R*_isobutane_) and the total separation factor (*β*) (isobutane over all other components) reached 87% and 1329, respectively. For comparison, the separation results obtained by the Chinese refinery with the distillation method and similar feed gas compositions are also shown in Table 1. The isobutane purity in the top gas was only 98.56 mol%, less than the 99.46 mol% purity of the slurry method. On the other hand, the isobutane concentration in the bottom product was as high as 36.04%, which resulted in a significant reduction in the isobutane yield (*R*_isobutane_: 54%). Additionally, the height and tray number of the distillation column were 72 m and 120, respectively, in order to meet the separation requirements. In this case, the equipment investment was expensive. Additionally, the reflux ratio of the distillation column was set to >10, which led to high energy costs, while there was no reflux in the slurry method. In summary, the slurry method provided great improvements relative to the traditional distillation method in separation efficiency, energy cost, and equipment investment.

**Table 1:** Operating conditions of the pilot plant and a comparison of the separation results between the pilot plant and a Chinese refinery using distillation method for the C4 mixtures with similar composition

Operating conditions		Sorption column		Desorption column		Slurry flow rate		Gas flow rate	
		~30 °C/2 bar		~60 °C/0.1 bar		12 L/h		150 L/h	
		Iso-butane	n-butane	Trans-2-butene	1-butene	Iso-butylene	Cis-2-butene	1,3-butadiene	
Pilot plant	Feed gas (mol%)	51.19	13.47	11.96	16.33	0.08	6.85	0.12	*R*_isobutane_ 87% *β* :1329
	Product gas (mol%)	99.46	0.32	0.03	0.13	0.02	0.04	0	
	Desorbed gas (mol%)	12.17	24.62	18.92	31.21	0.15	12.70	0.23	
Distillation	Feed gas (mol%)	54.59	12.78	7.61	14.43	0.13	10.37	0.09	*R*_isobutane_ 54%
	Top product (mol%)	98.56	0	0	1.31	0.03	0	0.10	
	Bottom product (mol%)	36.04	18.07	11.29	18.28	0.16	16.14	0.08	

*β*: separation factor (iso-butane over all other components).

*R*_isobutane_: recovery ratio of iso-butane.

The excellent stability of the slurried adsorbent can also be guaranteed because the slurry used in these experiments was investigated in the pilot plant for more than 7 months in flowing or static processes conducted within a temperature range of −10 to 70 °C. The uniform state of the recovered slurry (Supplementary Fig. 8c) and the XRD results for the recovered ZIF-8 powder (Supplementary Fig. 8a) also confirmed the structural integrity of ZIF-8 and the compatibility of ZIF-8 with solvent. These results were consistent with previous laboratory results. After these experiments, we removed a piece of packing from the sorption column after washing with water. As shown in Supplementary Fig. 8b, there was no slurry blockage or corrosion on the surfaces or insides of the stainless-steel structured packing, which meant that the slurry containing the solid phase could be used in a packed column for gas separation. The pilot test results provided powerful support for the further industrial application of porous slurries.

Separations of C4 olefins are also important and highly challenging in the petrochemical industry. To probe the use of the ZIF-8 slurry in this field, we preliminarily investigated the sorption and single-stage separation behaviours of five C4 olefins, isobutene, 1-butene, trans-2-butene, cis-2-butene, and 1,3-butadiene, with a ZIF-8/DMPU-water slurry containing a ZIF-8 fraction of 30 wt% and a water content of 20 wt% in the mixed solvent. The experimental results are presented in Supplementary Fig. 9 and Supplementary Tables 6–8. As shown in Supplementary Fig. 9, there were significant differences seen for both the sorption isotherms and sorption speeds of isobutene and those of the other four C4 olefins; this indicated potential for separating isobutene from the other four C4 olefins without branching CH_3_ groups by using this slurry, although this was not as pronounced as the prospect for separating n-butane/isobutane. However, these differences among the four C4 olefins without branching CH_3_ groups were disappointing. As shown in Supplementary Tables 6–8, the separation factors for isobutene over the other four C4 olefins, 1-butene, trans-2-butene, cis-2-butene, and 1,3-butadiene, ranged from 21 to 126, implying promise for separating isobutene from the other C4 olefins with this ZIF-8 slurry. 1-Butene seemed to be another exception; its selectivities for separation from trans-2-butene, cis-2-butene, and 1,3-butadiene were obviously larger than those of the other pairs trans-2-butene/cis-2-butene, trans-2-butene/1,3-butadiene, and cis-2-butene/1,3-butadiene. For example, the selectivity for separation of 1-butene from 1,3-butadiene reached 3.4; this was significantly higher than those for trans-2-butene, cis-2-butene, and 1,3-butadiene, which ranged from 1.1 to 1.6. More encouragingly, we found that as with the case of butane isomers, the selectivity for separation of 1-butene from 1,3-butadiene was further increased to 6.8 by increasing the water content in the mixed solvent to 60 wt% (see Supplementary Table 9). Hence, it is also promising to separate 1-butene from 1,3-butadiene by using the ZIF-8/DMPU-water slurry, and the separations of C4 olefins deserve further study. Finally, column breakthrough experiments were performed with two multicomponent C4 olefin gas mixtures, and the experimental results are plotted in Supplementary Fig. 10. As expected, isobutene showed the shortest breakthrough time. The second component was 1-butene, but the other components were difficult to distinguish from each other.

## Discussion

Traditional adsorption-based gas separation techniques, such as pressure (vacuum) swing adsorption (P(V)SA) and temperature swing adsorption (TSA), are more energy efficient than distillation for the separation of butane isomers. However, these techniques have inherent drawbacks. For example, there will inevitably be crushing of adsorbents and loss of pressure inside the adsorption column^51^. Shaped nonfluid solid-phase adsorbents are typically used with fixed beds in batch processes exhibiting low efficiencies. The slurry approach proposed in this work provides a potential solution to these problems by fluidizing the solid-phase adsorbents. On the other hand, the higher n-butane uptake seen with ZIF-8 at lower pressure, as shown in Fig. 2a, and the high n-butane desorption heat (~20–32 kJ/mol^31^) indicated that a TSA, but not a PSA, technique should be adopted when using ZIF-8 as an adsorbent. Heat transfer and heat integration would be challenging issues for a fixed bed, as heat conductivity is low for a porous medium. The slurry approach will become promising in this case because the cooling and heating of the slurry are easily carried out because the slurry flows like fluid. At the same time, vaporization of the water solvent, which has a low boiling point, in the desorption process would enhance the degassing efficiency due to the stripping effect of the water vapour.

ZIF-8 showed superior properties in terms of n-butane uptake and n-butane/isobutane selectivity. A carefully chosen ZIF-8/DMPU combination led to significantly faster sorption but an increase in isobutane sorption, thereby reducing the overall selectivity. The proposed slurry with the mixed DMPU-water solvent and a satisfactory sorption speed solved this problem by reducing the solubility of isobutane. Theoretically, solid ZIF-8 should have the highest sorption speed because it has the smallest mass transfer resistance, but this was not the case. The faster sorption speed of the slurry was attributed to dispersion of ZIF-8 by the DMPU solvent, which was confirmed by experimental evidence. First, the kinetic curves for the fresh and regenerated ZIF-8(30 wt%)/DMPU-water slurries (Supplementary Fig. 11a) showed that the regenerated slurry had a faster sorption speed, which would not have happened if the properties of the slurry had not changed. To confirm the dispersion state of ZIF-8 in the slurry, we measured the particle sizes of ZIF-8 in different slurries. As shown in Supplementary Fig. 11b, the average particle sizes of fresh ZIF-8/DMPU slurries and regenerated ZIF-8/DMPU-water slurries were ~2.2 and ~1.18 μm, respectively, while those of the fresh or regenerated ZIF-8/water slurry and fresh ZIF/DMPU-water slurry exceeded the upper limit (6 μm) of the measuring apparatus; this confirmed good dispersion of the ZIF-8 particles by the DMPU solvent. The SEM images for fresh and recovered ZIF-8 from the ZIF-8/DMPU-water slurry visually showed the differences in their surface morphologies (Supplementary Fig. 12). Agglomeration of ZIF-8 particles was significantly inhibited by the solvent DMPU. Agglomeration of ZIF-8 nanoparticles, which is favoured by the high surface energies, decreases the sorption speed by increasing the mass transfer resistance. This also explains why the sorption speed of solid ZIF-8 was reduced after shaping. In this case, the slurry method has more potential advantages than the fixed bed process because the kinetic performance of the slurry gradually improves over time.

It is very interesting that the separation ability of the ZIF-8/DMPU-water slurry was greater than those of the ZIF-8/water slurry and the ZIF-8/DMPU slurry. Mixing water and the DMPU solvent resulted in a significant synergic effect, and we hypothesize that there are three aspects of the mechanism for this synergic effect. First, the addition of DMPU to water in preparing the ZIF-8/water slurry increased the sorption speed of n-butane; fast prefilling of n-butane molecules into the pores of ZIF-8 inhibited subsequent adsorption of isobutane molecules^14^ because this is a very slow process even for pure isobutane, as demonstrated by Fig. 3b. The pressure profiles plotted in Supplementary Fig. 13 also support this mechanism; there was no obvious and continuous decline in the pressure of pure isobutane for the ZIF-8/water or the ZIF-8/DMPU-water slurry after a certain time period as depicted in Supplementary Fig. 3b, when n-butane sorption reached equilibrium. Second, the addition of water to the ZIF-8/DMPU slurry dramatically decreased the solubility of isobutane in the solvent and then raised the apparent selectivity of the slurry for n-butane over isobutane. In fact, this effect was expected from the single gas sorption experimental results shown in Fig. 3. Third, Li et al. found that hydrophilic solvent molecules assembled semipermeable films surrounding hydrophobic ZIF-8 particles, and this film provided selective hindrance for entry into the pores of ZIF-8 by gas molecules^52^. It is reasonable to believe that the structures and permeation selectivities of these films vary with solvent composition. Perhaps, it is more difficult for isobutane to penetrate the film assembled by water and DMPU together than to penetrate the films assembled by water or DMPU separately. This semipermeable film mechanism could also explain why the increased water content increased the selectivity for separation of 1,3-butadiene over 1-butene, as shown in Supplementary Table 9. Supplementary Fig. 14 shows a comparison of the sorption profiles for 1-butene and 1,3-butadiene with two ZIF-8/DMPU-water slurries containing different water contents, 20 and 60 wt%. The differences in the sorption speeds for 1-butene and 1,3-butadiene were enlarged drastically and then increased the kinetic separation selectivities remarkably when the water content in the mixed solvent was increased from 20 to 60 wt%. This enhancement effect could reasonably be attributed to increases in the permeation selectivities of the films assembled by solvent molecules around the ZIF-8 particles, although further research is needed. Tunable permeation selectivities for films assembled by solvent molecules may provide a way to separate close-boiling gas mixtures such as C4 olefins efficiently.

Overall, the excellent selectivity seen for n-butane over isobutane, in combination with the high sorption capacity and high sorption speed seen for n-butane, make the slurry approach proposed in this study a promising candidate for challenging separations of butane isomers.

## Methods

### Materials

Analytical grade solvents N,N-dimethylpropyleneurea (DMPU), carboxymethyl cellulose sodium (CMC), and hydroxyethyl cellulose (HEC) were purchased from Shanghai Aladdin BioChem Technology Co., Ltd. Tap water was used. n-Butane (99.5%) and isobutane (99.5%) were purchased from Beijing HaiPu Gases Industry Co., Ltd., and used to prepare n-butane/isobutane feed gas mixtures with different compositions. ZIF-8 was synthesized in our laboratory^50^, and the syntheses can be found in the Supplementary methods.

### Sorption measurements

Measurements of gas-slurry (solid) phase equilibria and kinetics, as well as mixed gas separations with different systems, were conducted with the apparatus shown in Supplementary Fig. 15. As shown in our previous reports^39,46^, there were two main components: a stainless steel blind cell with an effective volume of 132.4 cm^3^ (including the connected pipeline) and a transparent sapphire cell with an effective volume of 59.9 cm^3^ (including the connected pipeline). Both components were installed in a constant temperature air bath to maintain a uniform temperature. The maximum working pressure of two cells was designed to be 20 MPa. The temperature and pressure of the system were measured with a secondary platinum resistance thermometer (Pt100 type) and a differential pressure transducer, which exhibited uncertainties of ±0.1 K and ±0.002 MPa, respectively. An LG100H luminescence source was installed in the air bath so that phenomena occurring in the transparent sapphire cell could be observed more clearly. Real-time readings of pressure and temperature were automatically recorded with a computer. Additionally, the compositions of the n-butane/isobutane mixtures were analyzed by a Hewlett-Packard gas chromatograph (HP 7890).

Before each experiment, the sapphire cell was removed from the apparatus, thoroughly cleaned with tap water, and dried. The desired quantity of the solid-phase ZIF-8 or ZIF-8 slurry was weighed and added to the sapphire cell. The mixture of solid-phase ZIF-8 and liquid solvent was stirred to form a fine slurry. Subsequently, the cell was reinstalled into the air bath and fixed. The whole system (blind cell + sapphire cell + connected pipeline) was evacuated to remove air, and feed gas was injected into the blind cell with sufficiently high pressure from a gas cylinder. The air bath was powered on after setting the temperature to a given value. Once both the temperature and pressure of the blind cell were stable for one hour, the real-time pressure of gas in the blind cell was recorded as ${P}_{0}^{b}$. Then, the top valve of the sapphire cell was slowly opened to let the feed gas flow into the sapphire cell until the desired pressure value (*P*_0_) was reached, and magnetic stirring was established with a fixed rate to promote gas-slurry mass transfer. When the system pressure remained stable for 1 h (for gas‒liquid equilibrium sorption) or the separation reached the set time (for nonequilibrium kinetic separation), the magnetic stirrer was turned off, and the pressures of the blind cell and the sapphire cell were recorded as ${P}_{1}^{b}$ and *P*_E_, respectively. For separations of mixed gases, the n-butane/isobutane mixture after sorption separation in the sapphire cell was sampled with constant pressure by pushing the hand pump continuously and analyzed by using the HP 7890 gas chromatograph.

In this work, the uptake of each gas species in the slurry (or ZIF-8 powder) was calculated based on the mass balance, as follows.

The total moles of feed gas injected into the sapphire cell (*n*_*t*_) from the blind cell was calculated by the following equation:

1 $${n}_{t}=\frac{{P}_{0}^{b}{V}_{b}}{{Z}_{0}{RT}}-\frac{{P}_{1}^{b}{V}_{b}}{{Z}_{1}{RT}}$$

where ${P}_{0}^{b}$ and ${P}_{1}^{b}$ are the pressures of the blind cell before and after injecting the gas into the sapphire cell, respectively, *V*_*b*_ is the total volume of the blind cell together with the connected pipeline, *T* and *R* are the system temperature, and the universal gas constant, respectively, and the compressibility factors *Z*_0_ and *Z*_1_ were calculated with the BWRS equation of state at ${P}_{0}^{b}$ and ${P}_{1}^{b}$.

The moles of gas remaining in the gas phase of the sapphire cell after the sorption process were determined by:

2 $${n}_{E}=\frac{{P}_{E}{V}_{g}}{{Z}_{E}{RT}}$$

where *P*_*E*_, *V*_*g*_, and *Z*_*E*_ are the pressure, volume, and compressibility factor, respectively, of the gas phase in the sapphire cell after the sorption process.

The moles of (1) n-butane and (2) isobutane ab(d)sorbed by the slurry (or ZIF-8 powder) were calculated as follows:

3 $${n}_{1}={n}_{t}\times {z}_{1}-{n}_{E}\times {y}_{1}$$

4 $${n}_{2}={n}_{t}\times {z}_{2}-{n}_{E}\times {y}_{2}$$

where *z*_1_ and *z*_2_ are the mole fractions of n-butane and isobutane in the feed gas, respectively, and *y*_1_ and *y*_2_ are the mole fractions of n-butane and isobutane in the gas phase of the sapphire cell at equilibrium, respectively.

Therefore, the dry-basis mole fractions of n-butane (1) and isobutane (2) in the slurry (or ZIF-8 powder) were obtained by

5 $${x}_{1}=\frac{{n}_{1}}{{n}_{1}+{n}_{2}}$$

6 $${x}_{2}=\frac{{n}_{2}}{{n}_{1}+{n}_{2}}$$

In the gas separation process, a separation factor was defined as *β* to characterize the separation efficiency of the separation medium.

7 $${{\beta }}=\frac{{x}_{1}/{y}_{1}}{{x}_{2}/{y}_{2}}$$

The initial gas-slurry (solid) volume ratio was determined by

8 $${{\varphi }}=\frac{22.4{n}_{t}}{{V}_{l(s)}}$$

where *V*_*l*_ and *V*_*s*_ are the volumes of the slurry and ZIF-8 powder, respectively. The volume of the slurry in the sapphire cell was calculated indirectly by measuring its density and mass. The volume of the ZIF-8 powder is the ratio of its mass to the skeleton density (0.9244 g/cm^3^).

The sorption capacities (i.e., solubilities) of n-butane (1) and isobutane (2) in the slurry were calculated by

9 $${S}_{i}=\frac{{n}_{i}}{{V}_{l}}$$

Similarly, the uptakes for n-butane (1) and isobutane (2) by the ZIF-8 powder were determined with

10 $${S}_{i}=\frac{{n}_{i}}{m}$$

where *m* is the mass of the ZIF-8 powder.

The sorption coefficients of n-butane (1) and isobutane (2) in the slurry (or ZIF-8 powder) were calculated by

11 $${S}_{{Ci}}=\frac{{S}_{i}}{{P}_{E}\times {y}_{i}}$$

The removal ratio of n-butane (1) (*R*_1_) was used to indicate the capture ability of the slurry (or ZIF-8 powder) and was calculated by

12 $${R}_{1}=\left(1-\frac{{n}_{E}\times {y}_{1}}{{n}_{t}\times {z}_{1}}\right)\times 100\%$$

Methods for the breakthrough experiments and pilot-scale continuous separation experiments can be found in the Supplementary methods.

## Supplementary information

Supplementary Information

## Data Availability

All data generated or analyzed during this study are available within the paper and its supplementary information. Raw data are available from the Source Data file or corresponding authors upon request. Source data are provided with this paper.

## Source data

Source Data
