# Supplementary material for: Efficient separation of butane isomers via ZIF-8 slurry on laboratory- and pilot-scale
Source: Nat Commun. 2022 Aug 15;13:4792. doi: 10.1038/s41467-022-32418-6 (PMC9378693; doi:10.1038/s41467-022-32418-6)
Supplement: Supplementary file 1 — Supplementary Information [file 41467_2022_32418_MOESM1_ESM.pdf]

**Supplementary Information**

# Efficient separation of butane isomers via ZIF-8 slurry on laboratory- and pilot-scale

Mingke Yang[1], Huishan Wang[1], Julian Y. Zuo[2], Chun Deng[1], Bei Liu[1], Liya Chai[1], Kun Li[1], Han Xiao[1,3], Peng Xiao[1], Xiaohui Wang[1], Wan Chen[1], Xiaowan Peng[1], Yu Han[1], Zixuan Huang[1], Baocan Dong[1], Changyu Sun[1*], Guangjin Chen[1*]

[1]State Key Laboratory of Heavy Oil Processing, China University of Petroleum, Beijing 102249, China

[2]FMG Inc., Edmonton, Alberta T6N 1M9, Canada

[3]CenerTech Tianjin Chemical Research and Design Institute Co., Ltd., Tianjin 300131, China

[*]E-mail: cysun@cup.edu.cn (Changyu Sun); gjchen@cup.edu.cn (Guangjin Chen).

## Supplementary Figures

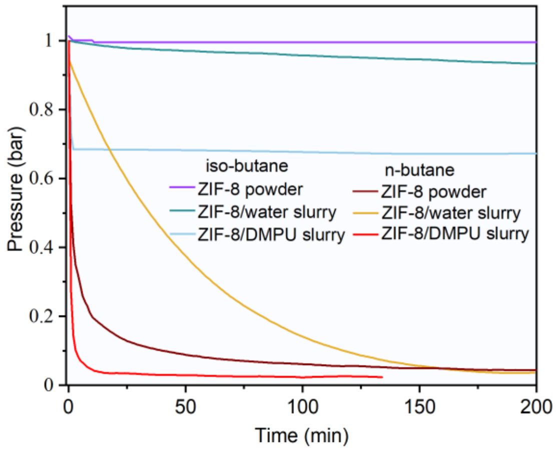


**Supplementary Figure 1.** The pressure variation of iso-butane and n-butane with the elapsed time on ZIF-8 powder, ZIF-8 (35wt%)/DMPU slurry and ZIF-8 (25wt%)/water slurry, where temperature, initial pressure, initial gas-slurry volume ratio, initial gas-solid volume ratio were set to 293.15 K, ~1 bar, ~6.8, ~26.7, respectively.

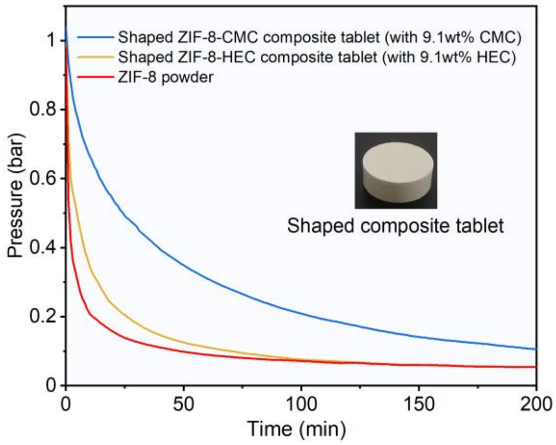


**Supplementary Figure 2.** The pressure variation of n-butane with the elapsed time on ZIF-8 powder and shaped ZIF-8 composite tablets at 293.15 K and an initial pressure of about 1 bar, where the mass of ZIF-8 in composite tablets is consistent with that of pure ZIF-8 powder. Carboxymethyl Cellulose Sodium (CMC) and Hydroxyethyl Cellulose (HEC) are two common adhesives.

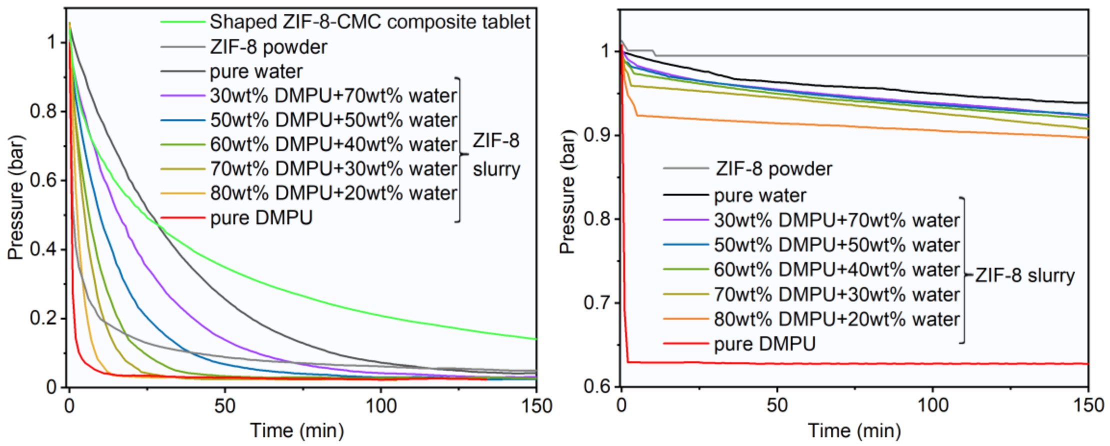


**Supplementary Figure 3.** The pressure variation of n-butane **(a)** and iso-butane **(b)** with the elapsed time on ZIF-8 powder, shaped ZIF-8 tablet and ZIF-8 slurries with different liquid phase composition, where temperature, initial pressure, initial gas-slurry volume ratio, initial gas-solid volume ratio and solid ZIF-8 content in the slurries were set to 293.15 K, ~1 bar, ~6.8, ~26.7 and 30 wt%, respectively.

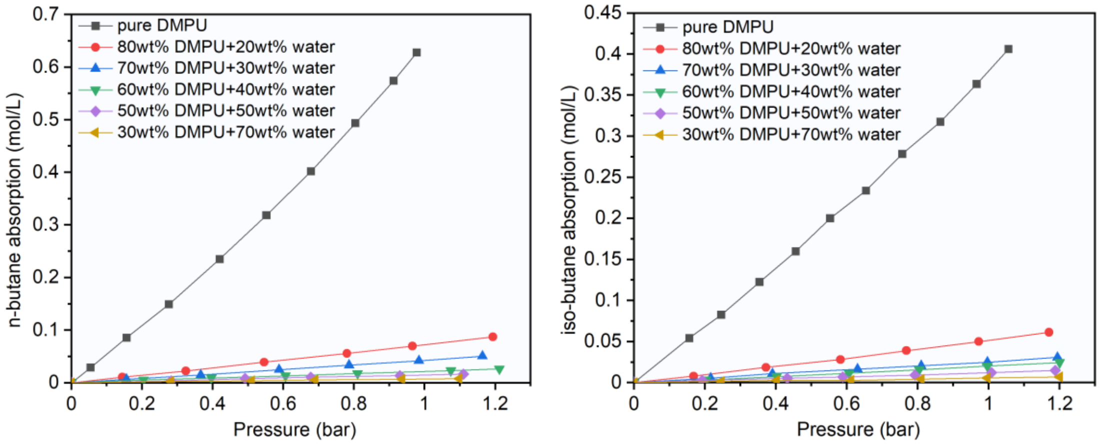


**Supplementary Figure 4.** Absorption isotherms of n-butane **(a)** and iso-butane **(b)** in the DMPU-water mixed solvents with different water content at 293.15 K.

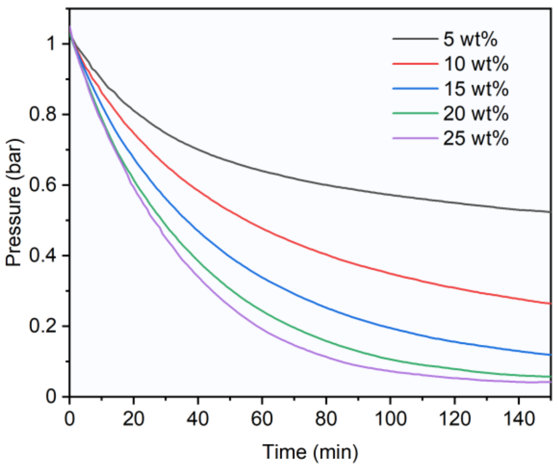


**Supplementary Figure 5.** The pressure variation of n-butane with the elapsed time on ZIF-8/water slurry with different ZIF-8 mass fractions at an initial pressure of about 1 bar and 293.15 K.

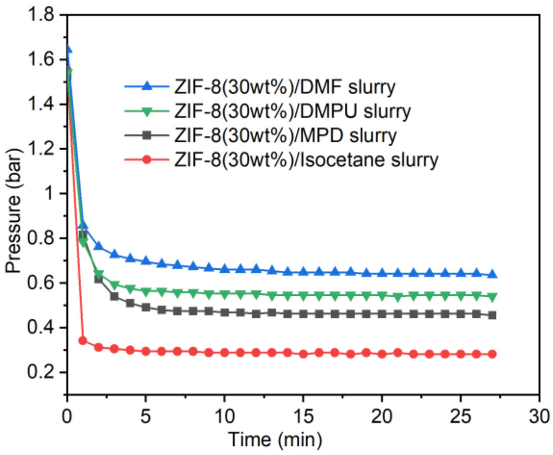


**Supplementary Figure 6.** The pressure variation of n-butane (1)/iso-butane (2) gas mixtures ($z_2$ = ~52 mol%) with the elapsed time on ZIF-8 (30 wt%) slurry with different solvents at an initial pressure of about 1.6 bar and 293.15 K. DMF: N,N-Dimethylformamide; MPD: 2-Methyl-2,4-pentanediol.

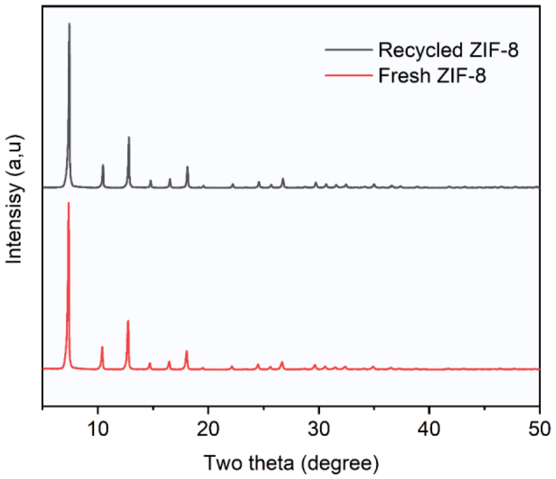


**Supplementary Figure 7.** X-ray diffraction of fresh ZIF-8 and ZIF-8 recovered from ZIF-8 (30 wt%) slurry with solvent composition of 70 wt% DMPU + 30 wt% water after 23 cycle sorption-desorption experiments.

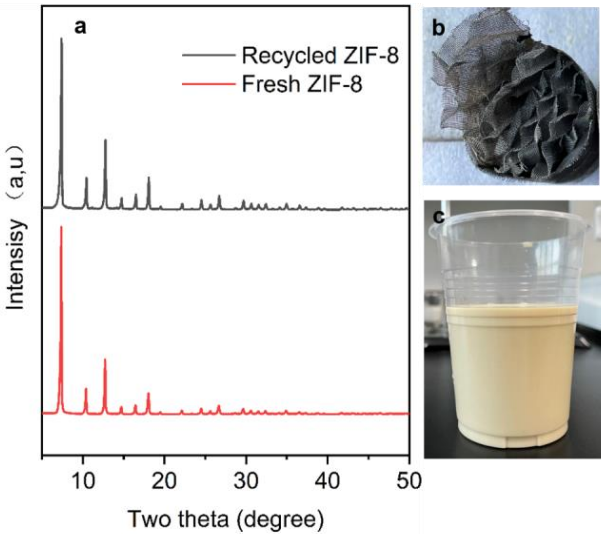


**Supplementary Figure 8. (a)** X-ray diffraction of fresh ZIF-8 and ZIF-8 recovered from the slurry after being used for 7 months in the pilot plant. Photographs of **(b)** CY700 structured packing in sorption column after water washing and **(c)** sampled ZIF-8 slurry with stable state after 7 months of use in the pilot plant.

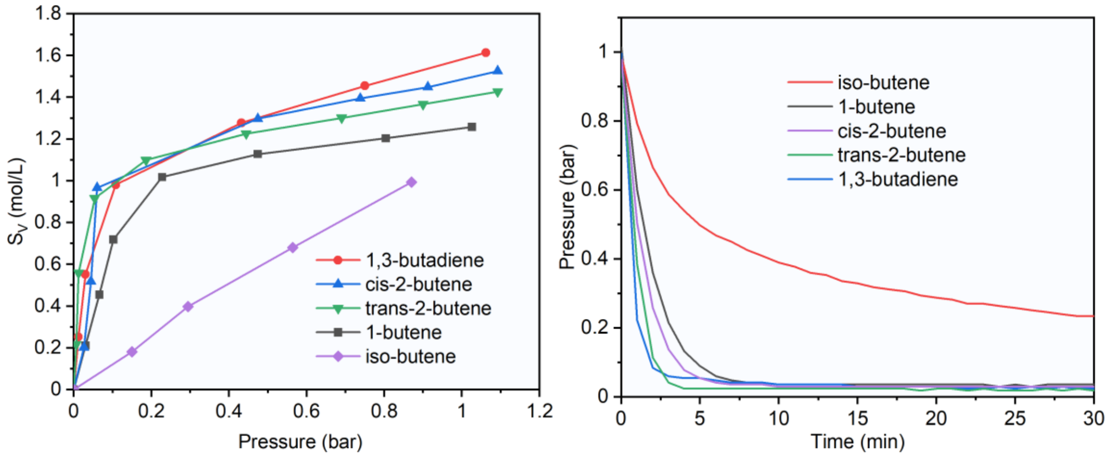


**Supplementary Figure 9. (a)** Sorption isotherms of C4 olefins on ZIF-8 (30wt%)/DMPU-water slurry at 293.15 K; **(b)** the pressure variation of C4 olefins with the elapsed time on ZIF-8(30wt%)/DMPU-water slurry at an initial pressure of about 1 bar and 293.15 K.

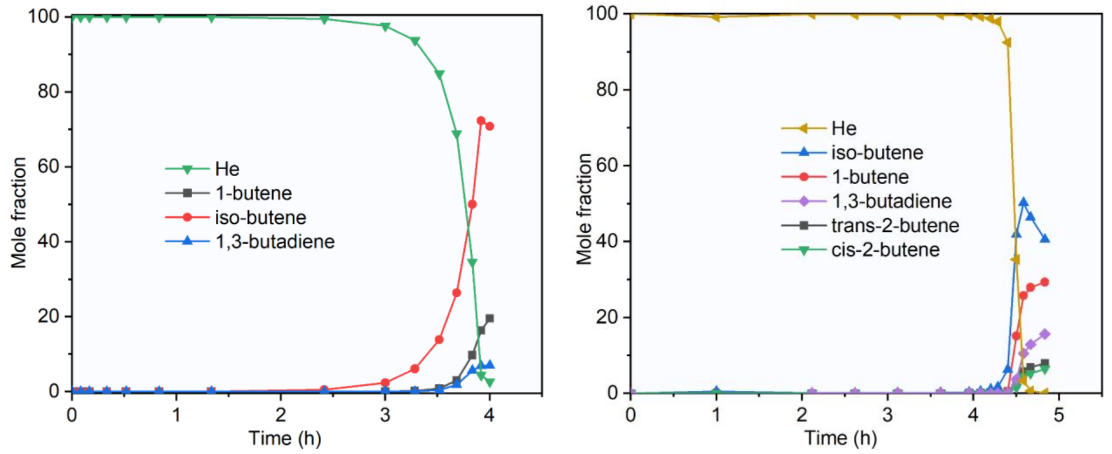


**Supplementary Figure 10.** Breakthrough curves of **(a)** three-component C4 olefin gas mixture [iso-butene(35.59 mol%)/1-butene(32.73 mol%)/1,3-butadiene(31.68 mol%)] and **(b)**: five-component C4 olefin gas mixture [iso-butene (26.24 mol%)/1-butene (27.19 mol%)/cis-2-butene (12.10 mol%)/1,3-butadiene (22.41 mol%)/ trans-2-butane (12.06 mol%)] in ZIF-8 (30wt%)/DMPU-water slurry under the condition of 303.15 K, 1 bar and inlet flow rate of 100 mL/min.

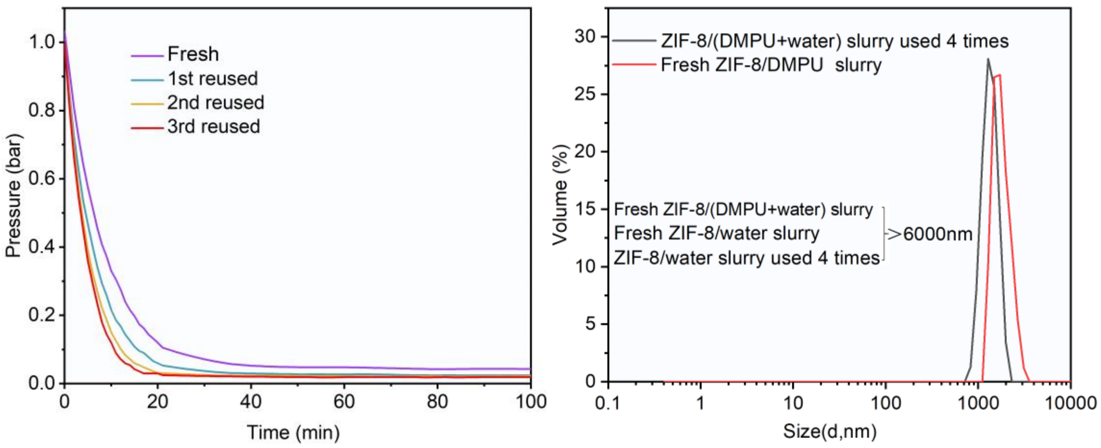


**Supplementary Figure 11. (a)** The pressure variation of n-butane with the elapsed time in fresh and regenerated ZIF-8(30wt%)/DMPU-water slurry at an initial pressure of about 1 bar and 293.15 K, **(b)** ZIF-8 particle size distribution curves in different slurries. The particle size of fresh ZIF-8/DMPU-water slurry, fresh ZIF-8/water slurry and ZIF-8/water slurry used 4 times exceed the upper limit of the instrument range (6000 nm). The ZIF-8 content in different slurries is 8 wt%.

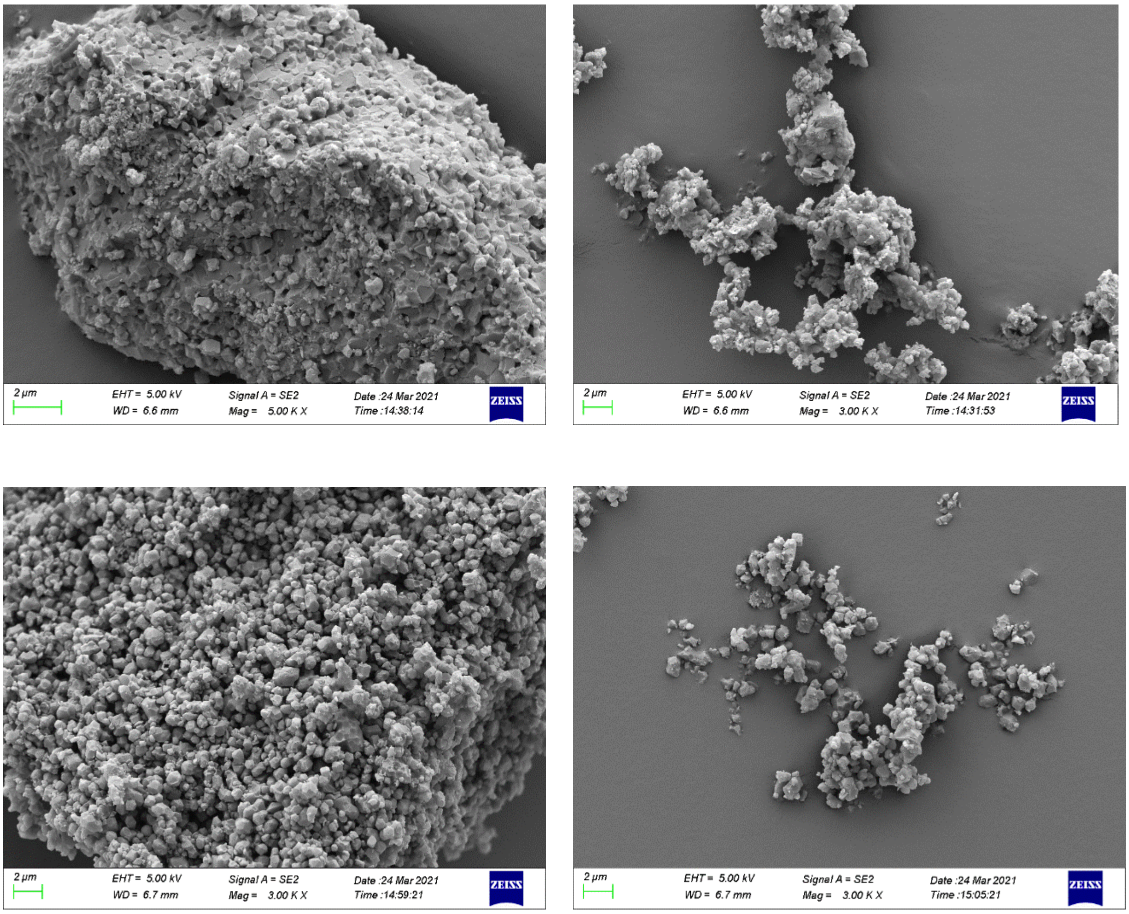


**Supplementary Figure 12.** Comparison of the SEM images of ZIF-8 powder between **(a, b)** fresh and **(c, d)** recovered from ZIF-8(30wt%)/DMPU-water slurry after being used for 4 times.

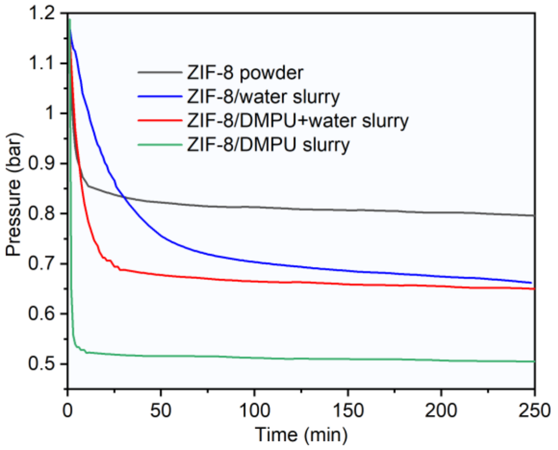


**Supplementary Figure 13.** The pressure variation of n-butane(1)/iso-butane(2) gas mixture ($z_{2}$ = 61.8 mol%) with the elapsed time on ZIF-8 powder, ZIF-8(25wt%)/water slurry, ZIF-8(30wt%)/DMPU-water slurry and ZIF-8(35wt%)/DMPU slurry, where temperature, initial pressure, initial gas-slurry volume ratio and initial gas-solid volume ratio for ZIF-8 powder were specified at 293.15 K, ~1.2 bar, ~8.2, 26.4, respectively.

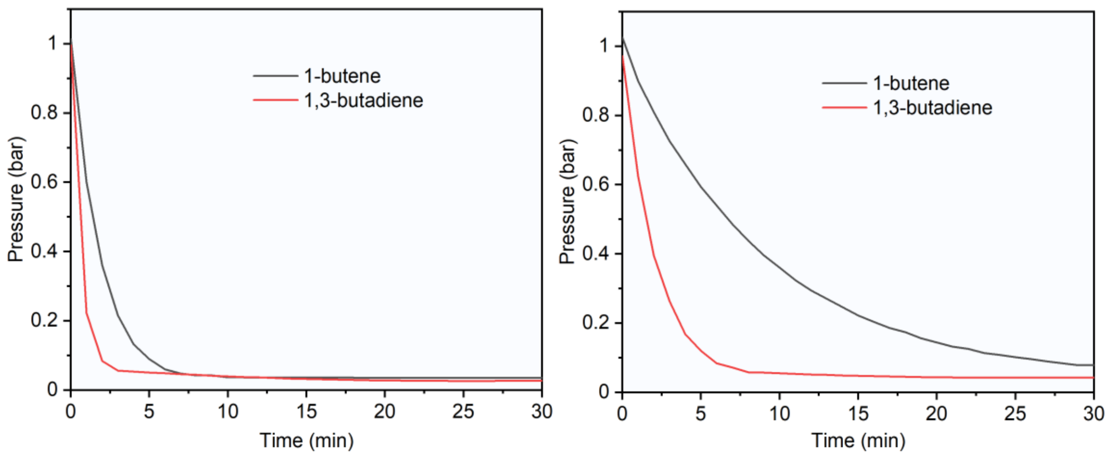


**Supplementary Figure 14.** Sorption kinetics comparison of 1-butene and 1,3-butadiene in ZIF-8(30 wt%) slurry with different solvent composition: **(a)** 80 wt% DMPU + 20 wt% water; **(b)** 40 wt% DMPU + 60 wt% water, where temperature, initial pressure, initial gas-slurry volume ratio were set to 293.15 K, ~1 bar, ~6.8, respectively.

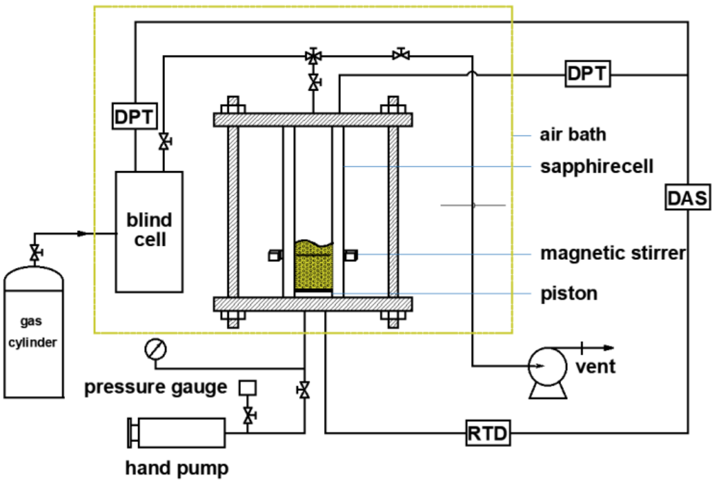


**Supplementary Figure 15.** Schematic diagram of experimental apparatus for phase equilibrium and kinetic experiments: DAS, data acquisition system; DPT, differential pressure transducer; RTD, resistance thermocouple detector.

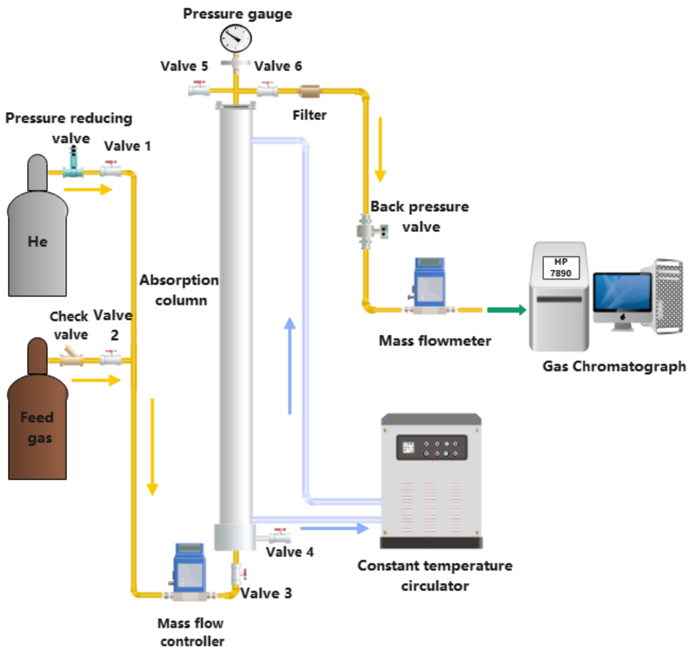


**Supplementary Figure 16.** Schematic diagram of the gas breakthrough experimental apparatus.

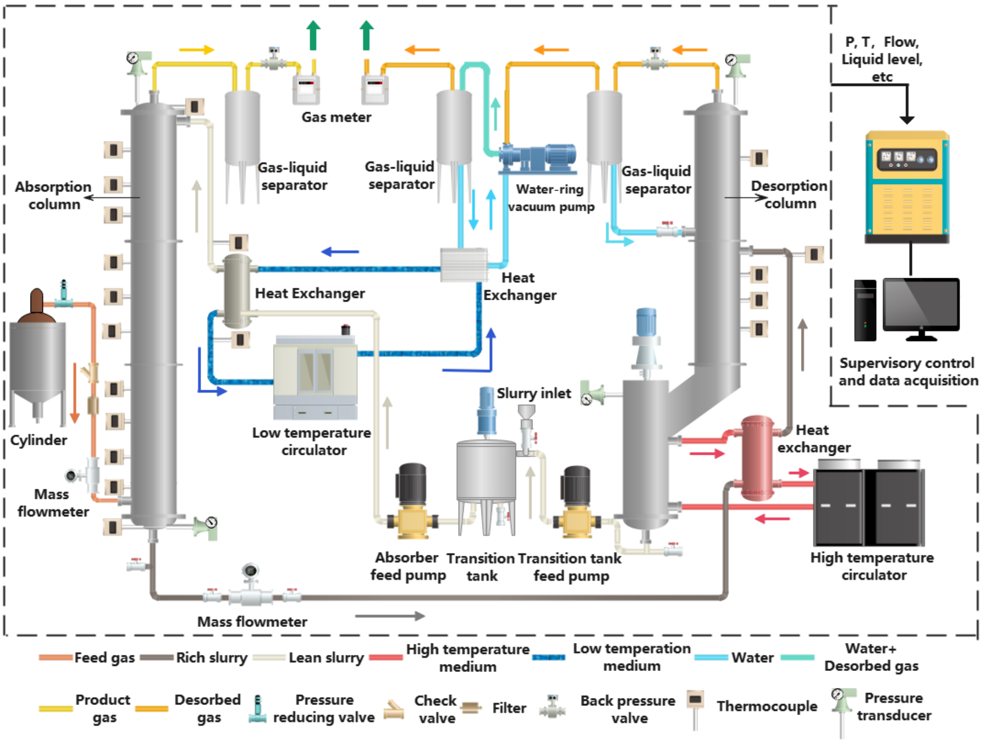


**Supplementary Figure 17.** Schematic diagram of the pilot plant for continuous C4 gas mixture sorption-desorption separation experiment.

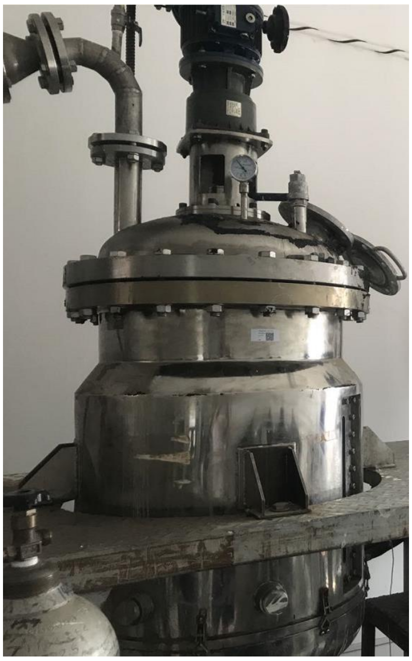

**Supplementary Figure 18.** ZIF-8 synthesis reactor

## Supplementary Tables

**Supplementary Table 1.** Separation results for n-butane(1)/iso-butane(2) gas mixture ($z_2$ = 61.8 mol%) using different sorbents at 293.15 K, where the ZIF-8 content of water-based, DMPU-based, and DMPU-water mixed-based slurries were set 25wt%, 35wt%, 30wt%, respectively.

| Sorbent | $\Phi$ | $P_E$(bar) | $y_2$(mol%) | $x_2$(mol%) | $S_{C1}$(**a**) | $S_{C2}$(**a**) | $R_1$(%) | $\beta$ |
|---|---|---|---|---|---|---|---|---|
| ZIF-8 powder | 39 | 0.97 | 98.33 | 0.5 | 39.881 | 0.003 | 97.3 | 11757 |
| ZIF-8/water slurry | 12 | 1.02 | 98.32 | 9.2 | 11.271 | 0.020 | 97.4 | 577 |
| ZIF-8/DMPU slurry | 19 | 0.74 | 98.20 | 32.6 | 23.648 | 0.151 | 97.9 | 113 |
| ZIF-8/80wt%DMPU+20wt%water | 15 | 1.04 | 98.65 | 7.6 | 17.345 | 0.019 | 97.9 | 890 |
| ZIF-8/70wt%DMPU+30wt%water | 14 | 1.02 | 98.97 | 6.3 | 21.819 | 0.016 | 98.3 | 1378 |
| ZIF-8/60wt%DMPU+40wt%water | 14 | 0.99 | 98.93 | 5.8 | 21.726 | 0.015 | 98.3 | 1494 |
| ZIF-8/50wt%DMPU+50wt%water | 14 | 1.02 | 98.85 | 4.9 | 20.050 | 0.012 | 98.2 | 1684 |
| ZIF-8/30wt%DMPU+70wt%water | 14 | 1.04 | 99.05 | 3.4 | 24.173 | 0.008 | 98.5 | 2985 |

$\Phi$: initial gas-slurry(solid) volume ratio; ***a*:** mmol/g/bar or mol/L/bar;
$z_i$, $y_i$, $x_i$ are the mole fractions of component i in feed gas, equilibrium gas and liquid phase (gas dry-basis composition), respectively.
$R_1$: the removal ratio of component i; $\beta$: separation factor.

**Supplementary Table 2.** Separation results for n-butane (1)/iso-butane (2) gas mixtures by using recycled ZIF-8 /DMPU-water slurry at 293.15 K and an initial gas-slurry volume ratio of about 20, where the ZIF-8 content in the fresh slurry and water content in the mixed solvent were both 30 wt%.

| Reused time | $z_2$(mol%) | $P_E$(bar) | $y_2$(mol%) | $x_2$(mol%) | $S_{C2}$ (mol/L/bar) | $R_1$ (%) | $\beta$ |
|---|---|---|---|---|---|---|---|
| 0 | 92.13 | 1.48 | 99.69 | 43.2 | 0.034 | 96.5 | 422 |
| 3 | 90.24 | 1.43 | 99.70 | 41.9 | 0.042 | 97.5 | 466 |
| 8 | 90.99 | 1.46 | 99.65 | 40.5 | 0.037 | 97.0 | 414 |
| 12 | 90.45 | 1.46 | 99.61 | 37.4 | 0.034 | 97.3 | 543 |
| 22 | 89.62 | 1.44 | 99.66 | 38.3 | 0.039 | 97.3 | 471 |

**Supplementary Table 3.** Separation results for n-butane(1)/iso-butane(2) gas mixtures by using recycled ZIF-8 /DMPU-water slurry at 293.15 K, an initial gas-slurry volume ratio of about 20 but different desorption conditions, where the ZIF-8 content in the fresh slurry and water content in the mixed solvent were both 30 wt%.

| Desorption conditions | | $z_2$ (mol%) | $P_E$(bar) | $y_2$ (mol%) | $x_2$ (mol%) | $S_{C2}$ (mol/L/bar) | $R_1$ (%) | $\beta$ |
|---|---|---|---|---|---|---|---|---|
| T (℃) | t (min) | | | | | | | |
| fresh | | 92.13 | 1.48 | 99.69 | 43.2 | 0.034 | 96.5 | 422 |
| 60 | 30 | 91.24 | 1.47 | 99.78 | 45.7 | 0.044 | 97.9 | 542 |
| 50 | 60 | 90.45 | 1.46 | 99.69 | 37.4 | 0.034 | 97.3 | 543 |
| | 30 | 94.07 | 1.48 | 99.81 | 57.1 | 0.046 | 97.3 | 400 |
| | 20 | 90.24 | 1.43 | 99.70 | 41.9 | 0.042 | 97.5 | 466 |
| | 10 | 91.49 | 1.44 | 99.63 | 48.9 | 0.048 | 96.3 | 281 |

**Supplementary Table 4.** Separation results for n-butane(1)/iso-butane(2) gas mixtures by using ZIF-8(30 wt%) slurry with different solvents at 293.15 K and an initial gas-slurry volume ratio of about 12.

| solvent | $z_2$(mol%) | $P_E$(bar) | $y_2$(mol%) | $x_2$(mol%) | $S_2$(mol/L) | $\beta$ |
|---|---|---|---|---|---|---|
| N,N-Dimethylformamide | 53.5 | 0.63 | 96.3 | 28.2 | 0.10 | 67 |
| DMPU | 51.2 | 0.54 | 97.7 | 27.4 | 0.11 | 114 |
| 2-Methyl-2,4-pentanediol | 53.5 | 0.46 | 96.3 | 36.0 | 0.14 | 46 |
| isohexadecane | 52.2 | 0.28 | 92.2 | 43.8 | 0.18 | 15 |

**Supplementary Table 5.** Outlet gas concentration results of the breakthrough test for n-butane(1)/iso-butane(2) gas mixture ($z_2$ = 59 mol%) using ZIF-8(30wt%)/DMPU-water slurry at 303.15K and 2 bar.(Gas flow rate: 60 mL/min)

| Time (h) | $y_{iso\text{-}butane}$ (mol%) | $y_{n\text{-}butane}$ (mol%) | $y_{He}$ (mol%) | $\frac{y_{iso\text{-}butane}}{y_{iso\text{-}butane}+y_{n\text{-}butane}}\times 100\%$ |
|---|---|---|---|---|
| 0.17 | 0.03 | 0 | 99.97 | 0 |
| 0.33 | 4.21 | 0.06 | 95.73 | 98.6189 |
| 0.50 | 22.05 | 0.16 | 77.80 | 99.3019 |
| 0.67 | 51.64 | 0.26 | 48.10 | 99.5029 |
| 0.83 | 70.66 | 0.26 | 29.09 | 99.6334 |
| 1.00 | 84.86 | 0.37 | 14.77 | 99.5647 |
| 1.33 | 94.87 | 0.43 | 4.70 | 99.5488 |
| 1.67 | 97.58 | 0.48 | 1.94 | 99.5085 |
| 2.00 | 98.50 | 0.58 | 0.92 | 99.4126 |
| 2.33 | 98.41 | 0.56 | 1.04 | 99.4372 |
| 2.67 | 98.53 | 0.51 | 0.96 | 99.485 |
| 3.25 | 98.84 | 0.55 | 0.61 | 99.4486 |
| 3.75 | 98.52 | 0.62 | 0.86 | 99.3776 |
| 4.33 | 98.70 | 0.76 | 0.54 | 99.2379 |
| 4.83 | 98.31 | 0.89 | 0.81 | 99.1078 |
| 5.33 | 98.13 | 1.07 | 0.80 | 98.9224 |
| 5.83 | 97.91 | 1.32 | 0.77 | 98.6748 |
| 6.33 | 97.77 | 1.48 | 0.76 | 98.5137 |
| 6.83 | 97.59 | 1.67 | 0.75 | 98.3185 |
| 7.42 | 97.16 | 2.41 | 0.43 | 97.5846 |
| 8.00 | 96.25 | 3.01 | 0.74 | 96.9645 |
| 8.30 | 95.68 | 3.60 | 0.72 | 96.375 |
| 8.75 | 95.01 | 4.59 | 0.40 | 95.3935 |
| 9.10 | 93.76 | 5.55 | 0.69 | 94.4143 |
| 9.38 | 93.03 | 6.59 | 0.39 | 93.3855 |
| 9.67 | 91.43 | 7.87 | 0.70 | 92.0758 |
| 9.92 | 90.44 | 9.18 | 0.38 | 90.788 |
| 10.12 | 89.25 | 10.40 | 0.36 | 89.5649 |

$y_{iso\text{-}butane}$ , $y_{n\text{-}butane,}$ $y_{He}$ are the concentration of iso-butane, n-butane and He in the outlet gas.

**Supplementary Table 6.** Separation results of 1-butene(1)/iso-butene(2) gas mixture ($z_2$=49.23 mol%) and 1,3-butadiene(1)/iso-butene(2) gas mixture ($z_2$=57.84 mol%) by using ZIF-8 (30 wt%)/DMPU-water slurry at 293.15 K.

| | Separation time (min) | $\Phi$ | $P_E$(bar) | $y_2$(mol%) | $x_2$(mol%) | $\beta$ |
|---|---|---|---|---|---|---|
| 1-butene/iso-butene | 27 | 25 | 0.59 | 86.94 | 17.55 | 31 |
| 1,3-butadiene/iso-butene | 15 | 25 | 0.66 | 96.16 | 17.38 | 119 |

**Supplementary Table 7.** Separation result of three-component C4 olefin gas mixture [iso-butene($z_1$=35.59 mol%)/1-butene ($z_2$=32.73 mol%)/1,3-butadiene($z_3$=31.68 mol%)] by using ZIF-8 (30 wt%)/DMPU-water slurry at 293.15 K.

| iso-butene(1)/1-butene(2)/1,3-butadiene(3) | | Separation time: 12 min | | $\Phi$: 26 | $P_E$: 0.53 bar |
|---|---|---|---|---|---|
| $y_1$: 80.23 mol% | $y_2$ 14.92 mol% | $y_3$: 4.85 mol% | $x_1$: 6.44 mol% | $x_2$: 44.36 mol% | $x_3$: 49.20 mol% |
| $\beta_{2\text{-}1}$=37 | | $\beta_{3\text{-}1}$=126 | | $\beta_{3\text{-}2}$=3.4 | |

$\beta_{i-j} = \frac{x_i / y_i}{x_j / y_j}$, where $x_i$ and $y_i$ are the mole fractions of component $i$ in the slurry and vapor phases, respectively.

**Supplementary Table 8.** Separation result of five-component C4 olefin gas mixture [iso-butene (26.24 mol%)/1-butene (27.19 mol%)/cis-2-butene (12.10 mol%)/1,3-butadiene (22.41 mol%)/trans-2-butene (12.06 mol%)] by using ZIF-8 (30 wt%)/DMPU-water slurry at 293.15 K.($x$, $y$: mol%)

| iso-butene(1)/1-butene(2)/cis-2-butene(3)/1,3-butadiene(4)/trans-2-butene(5) | | | | | | Separation time: 13 min | | $\Phi$: 32 | $P_E$: 0.59 bar |
|---|---|---|---|---|---|---|---|---|---|
| $y_1$:63.72 | $y_2$:19.55 | $y_3$: 6.38 | $y_4$: 6.88 | $y_5$: 3.47 | $x_1$: 4.76 | $x_2$:30.43 | $x_3$:18.32 | $x_4$:30.14 | $x_5$:16.35 |
| $\beta_{2\text{-}1}$:21 | $\beta_{3\text{-}1}$:38 | $\beta_{4\text{-}1}$:59 | $\beta_{5\text{-}1}$:63 | $\beta_{3\text{-}2}$:1.8 | $\beta_{4\text{-}2}$:2.8 | $\beta_{5\text{-}2}$:3.0 | $\beta_{4\text{-}3}$:1.5 | $\beta_{5\text{-}3}$:1.6 | $\beta_{5\text{-}4}$:1.1 |

$\beta_{i-j} = \frac{x_i / y_i}{x_j / y_j}$, where $x_i$ and $y_i$ are the mole fractions of component $i$ in the slurry and vapor phases, respectively.

**Supplementary Table 9.** Separation result for 1,3-butadiene/1-butene gas mixture ($z_2$=50.69 mol%) by using ZIF-8(30 wt%) slurry with solvent composition of 40 wt% DMPU + 60 wt% water at 293.15 K.

| | Separation time (min) | $\Phi$ | $P_E$(bar) | $y_2$(mol%) | $x_2$(mol%) | $\beta$ |
|---|---|---|---|---|---|---|
| 1,3-butadiene/1-butene | 8 | 30 | 0.61 | 77.06 | 33.19 | 6.8 |

## Supplementary Methods

**Breakthrough curve measurement.** The two-component gas breakthrough curves are measured by a dynamic breakthrough equipment, as shown in Supplementary Fig. 16. The main part of the equipment is a stainless-steel column (2.5 cm inner diameter × 160cm) with a jacket that is connected to a constant temperature circulator for temperature control. There is a kettle with a volume of about 1000 mL under the column, and a gas distributor is placed inside. The weight of the ZIF-8/DMPU-water slurry loaded in the column and the kettle is 1800 g, and no additional activation step for adsorbent is required. First, the column is purged with pure He for 1h; the temperature and pressure are maintained at 303 K and 2 bar, respectively. Then, the feed gas [n-butane(41mol%)/iso-butane(59 mol%)] at a flow rate of 60 mL/min is introduced. The outlet gas from the column is analyzed by a gas chromatograph (HP 7890, Agilent). After the breakthrough test, the rich slurry is regenerated by He purge (100 mL/min) at 353 K for 36 h.

**Pilot scale continuous separation experiment.** The continuous C4 mixed gas sorption-desorption separation apparatus with an explosion-proof function was designed and set up. The physical picture and the schematic diagram of the apparatus are shown in Fig. 7 and Supplementary Fig. 17. The sorption column (5.17 m) and the desorption column (5.48 m) with packing section are the main components of the experimental setup. The effective packing section of the sorption column has an inner diameter of 4.7 cm and a height of 3.75 m (1.25 m × 3). The effective packing section of the desorption column has an inner diameter of 9.8 cm and a height of 2.5 m (1.25 m × 2). The packing type of the two columns is CY700 structured packing made of 316 stainless steel.

Each experiment begins with vacuuming the sorption and desorption columns to 0.1 bar.

Then, the feed gas enters the bottom of the sorption column through a mass flow meter. When the sorption column reaches the desired pressure controlled by a back pressure valve, the lean ZIF-8 slurry is introduced into the sorption column at the top using a constant flow pump to contact with feed gas. The outlet gas at the top of the sorption column (product gas) is sampled and analyzed after passing through a gas-liquid separation tank and a gas meter. The rich ZIF-8 slurry from the outlet of the sorption column passes through a mass flow meter and a preheater, and finally enters the desorption column in the middle, driven by differential pressure between the two columns. The rich slurry is desorbed by heating together with vacuuming. A vacuum back pressure valve is used to control the vacuum degree of the desorption column. The bottom of the column (9.6 cm inner diameter × 2.98 m) without packing is equipped with an electromagnetic stirrer to prevent precipitation of the ZIF-8 slurry and a jacket that is connected to a high temperature circulator to maintain temperature at a desired value. After desorption, the desorbed gas and the lean slurry flow out from the top and bottom of the desorption column, respectively. In order to pump the slurry from the desorption column with negative pressure to the sorption column with positive pressure smoothly, an atmospheric transition tank is added between the two columns. The two columns are connected by the pipelines with double layers of insulation: a vacuum jacket and cotton insulation. A supervisory data-control-acquisition system is responsible for acquiring and displaying experimental data, such as temperature, pressure, flow rate, liquid level etc., and the control of equipment parameters. Each experiment lasts 9-10 h and when the system reaches a steady state, several samples are taken from the outlet for analysis.

Separation factor (*β*) of iso-butane (i) over other easily adsorbed components (e) and the recovery ratio of iso-butane ($R_{\text{iso-butane}}$) are two indicators for evaluating the separation efficiency of the pilot plant.

$$\beta = \frac{y_{out-i} / y'_{out-i}}{y_{out-e} / y'_{out-e}} \qquad \text{(Supplementary equation S1)}$$

where $y_{out-i}$ and $y'_{out-i}$ are the mole fractions of iso-butane at the outlet of the sorption column and the desorption column, $y_{out-e}$ and $y'_{out-e}$ are the sum of the mole fractions of all gases except iso-butane at the outlet of the sorption column and the desorption column.

$$R_{\text{iso-butane}} = \frac{V_{out} y_{out-i}}{V_{in} y_{in-i}} \times 100\% \qquad \text{(Supplementary equation S2)}$$

where $V_{in}$ and $V_{out}$ are the volumetric flow rates of the gas mixture at the inlet and outlet of the sorption column, $y_{in-i}$ is the mole fraction of iso-butane at the inlet of the sorption column. $V_{out}$ is calculated based on the mass balance of iso-butane as introduced below.

$$V_{in} y_{in-i} = V_{out} y_{out-i} + V'_{out} y'_{out-i} = V_{out} y_{out-i} + (V_{in} - V_{out}) y'_{out-i} \qquad \text{(Supplementary equation S3)}$$

where $V'_{out}$ is the volumetric flow rate of the gas mixture at the outlet of the desorption column.

$V_{out}$ can be obtained by Supplementary equation S3:

$$V_{out} = \frac{V_{in}(y_{in-i} - y'_{out-i})}{y_{out-i} - y'_{out-i}} \qquad \text{(Supplementary equation S4)}$$

Then, $R_{\text{iso-butane}}$ can be obtained by substituting Supplementary equation S4 into the Supplementary equation S2:

$$R_{\text{iso-butane}} = \frac{(y_{in-i} - y'_{out-i}) y_{out-i}}{(y_{out-i} - y'_{out-i}) y_{in-i}} \times 100\% \qquad \text{(Supplementary equation S5)}$$

**ZIF-8 synthesis.** The adsorbent ZIF-8 used in all experiments including pilot test in this paper was prepared by a green, low-cost, high-yield synthesis process developed by our research group[1]. Firstly, a solid mixture of 1172 g of 2-methylimidazole and 559 g of basic zinc carbonate ($ZnCO_3 \cdot 2Zn(OH)_2 \cdot H_2O$) was dissolved in 6327 g of solvent ethylene glycol and added to the synthesis reactor **(**Supplementary Fig. 18**)**. Then, the reaction gas $CO_2$ was continuously added into the synthesis reactor, and fully stirred until the equilibrium pressure reached 0.2 MPa. After 30 min of pressure stabilization, the synthesis reactor was vacuumed (0.01 MPa) at 333.15 K for 1 h. Subsequently, the product was collected by filtering and drying at 453.15 K for 12 h.

**Characterization.** ZIF-8 is characterized by a D8-Advanced X-ray diffractometer (Bruker, Germany) for the diffraction analysis under the condition of filtered Cu Ka radiation (λ=1.54 Å) at room temperature, accelerating voltage of 40 kV, tube current of 40 mA. The morphologies of the samples are obtained by a Gemini 300 scanning electron microscope (Zeiss, Germany). Particle size distribution of the slurry are measured by a Mastersizer 2000 laser diffraction instrument (Malvern, England).

**Supplementary Reference**


1. Chen G, Liu B, Li H, Yang M, Sun C, Chen W. Preparation method for zeolitic imidazolate frameworks. Patent No.: US 10,815,253 B2. (2020).
